# 1-Naphthyl 9*H*-carbazole-4-sulfonate

**DOI:** 10.1107/S1600536808016334

**Published:** 2008-06-07

**Authors:** R. Arulmozhi, Jasmine P. Vennila, Sunil Manohar Babu, Helen P. Kavitha, V. Manivannan

**Affiliations:** a Department of Chemistry, SRM University, Kattankulathur 603 203, Kanchipuram, India; b Department of Physics, Panimalar Institute of Technology, Chennai 600 095, India; c Nicholas Piramal Research Centre, Nicholas Piramal India Limited, Mumbai 400 063, India; d Department of Chemistry, SRM University, Ramapuram, Chennai 600 089, India; e Department of Physics, Presidency College, Chennai 600 005, India

## Abstract

In the title compound, C_22_H_15_NO_3_S, the plane of the carbazole ring system forms a dihedral angle of 65.06 (4)° with the naphthalene ring system. In the crystal structure, a weak intra­molecular C—H⋯O inter­action is observed between the naphthalene ring system and the sulfonate group. Two weak inter­molecular C—H⋯O inter­actions are also observed.

## Related literature

For biological activity, see: Itoigawa *et al.* (2000 ▶); Tachibana *et al.* (2001 ▶). For the structure of closely related compounds, see: Manivannan *et al.* (2005 ▶); Hosomi *et al.* (2000 ▶).
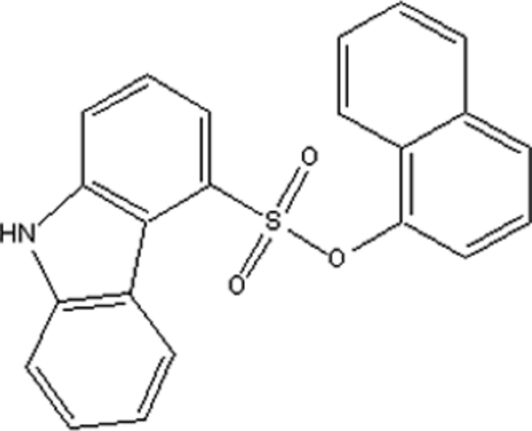


## Experimental

### 

#### Crystal data

C_22_H_15_NO_3_S
*M* *_r_* = 373.41
Orthorhombic, 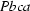

*a* = 14.2365 (5) Å
*b* = 9.2098 (4) Å
*c* = 26.3865 (10) Å
*V* = 3459.7 (2) Å^3^
*Z* = 8
Mo *K*α radiation
μ = 0.21 mm^−1^
*T* = 295 (2) K
0.20 × 0.16 × 0.14 mm

#### Data collection

Bruker Kappa APEXII diffractometer
Absorption correction: multi-scan (*SADABS*; Sheldrick, 1996 ▶) *T*_min_ = 0.959, *T*_max_ = 0.971
22287 measured reflections
4600 independent reflections
2928 reflections with *I* > 2σ(*I*)
*R*_int_ = 0.034

#### Refinement

*R*[*F* ^2^ > 2σ(*F* ^2^)] = 0.043
*wR*(*F* ^2^) = 0.144
*S* = 1.05
4600 reflections
244 parameters
H-atom parameters constrained
Δρ_max_ = 0.33 e Å^−3^
Δρ_min_ = −0.31 e Å^−3^

### 

Data collection: *APEX2*; cell refinement: *APEX2*; data reduction: *APEX2*; program(s) used to solve structure: *SHELXS97* (Sheldrick, 2008 ▶); program(s) used to refine structure: *SHELXL97* (Sheldrick, 2008 ▶); molecular graphics: *PLATON* (Spek, 2003 ▶); software used to prepare material for publication: *SHELXL97*.

## Supplementary Material

Crystal structure: contains datablocks I, global. DOI: 10.1107/S1600536808016334/is2296sup1.cif

Structure factors: contains datablocks I. DOI: 10.1107/S1600536808016334/is2296Isup2.hkl

Additional supplementary materials:  crystallographic information; 3D view; checkCIF report

## Figures and Tables

**Table 1:** Hydrogen-bond geometry (Å, °)

*D*—H⋯*A*	*D*—H	H⋯*A*	*D*⋯*A*	*D*—H⋯*A*
C2—H2⋯O2	0.93	2.42	2.835 (3)	107
C8—H8⋯O3^i^	0.93	2.50	3.403 (3)	164
C17—H17⋯O3^ii^	0.93	2.54	3.364 (3)	147

Symmetry codes: (i) 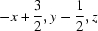; (ii) 
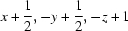
.

**Fig. 1.**
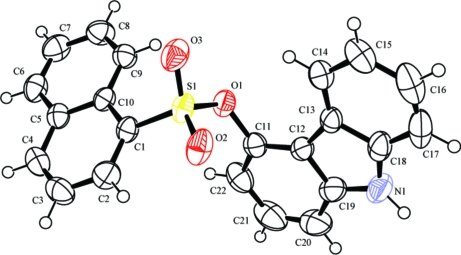
The molecular structure of (I), with atom labels and 50% probability displacement ellipsoids for non-H atoms.

**Fig. 2.**
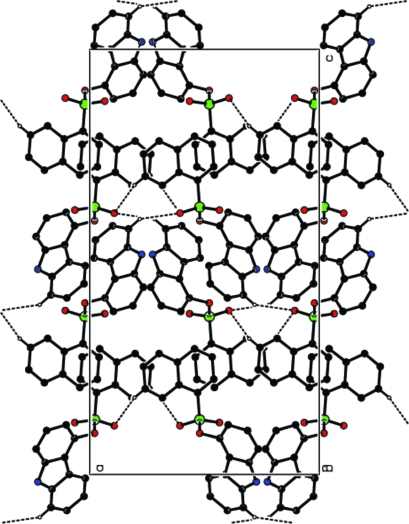
The packing of (I), viewed down the b axis. Hydrogen bonds are shown as dashed lines. H atoms not involved in hydrogen bonding have been omitted.

## supplementary crystallographic information

### Comment

Carbazole derivatives possess various biological activities, such as antitumor (Itoigawa *et al.*, 2000), antioxidative (Tachibana *et al.*, 2001). We report the crystal structure of the title compound, (I). The geometric parameters of the molecule of (I) (Fig. 1) agree well with the reported structures (Manivannan *et al.*, 2005; Hosomi *et al.*, 2000).

The plane of the carbazole ring forms a dihedral angle of 65.06 (4)° with the naphthalene ring. The torsion angles C2—C1—S1—O2 and C10—C1—S1—O3 [4.21 (19) ° and 46.22 (18)°, respectively] indicate a *syn* conformation of sulfonyl moiety. The molecular structure is stabilized by a weak intramolecular C—H···O interaction and the crystal packing is stabilized by weak intermolecular C—H···O interactions.

### Experimental

1-Naphthalene sulfonyl chloride (1.19 g, 5.2 mmol) dissolved in methelene dichloride was slowly added to 9H carbazol-4-ol (0.9 g, 4.8 mmol), followed by the addition of triethylamine (0.72 g, 7 mmol) at 20 °C. The reaction mixture was warmed at 40 °C and maintained at that temperature for 4 h. The reaction mixture was cooled to 15 °C. and mixed with 10 ml of methelene dichloride and 10 ml of water. The methelene dichloride layer was separated, washed to neutral pH with 5% aqueous sodium dicarbonate solution, dried over anhydrous sodium sulfate and concentrated. The crude compound was recrystallized from toluene.

### Refinement

H atoms were positioned geometrically (C—H = 0.93 and  N—H = 0.86 Å) and refined using riding model, with *U*_iso_(H) = 1.2*U*_eq_(C,N).

### Crystal data

**Table:**

C_22_H_15_NO_3_S	*F*_000_ = 1552
*M**_r_* = 373.41	*D*_x_ = 1.434 Mg m^−^^3^
Orthorhombic, *P**b**c**a*	Mo *K*α radiation λ = 0.71073 Å
Hall symbol: -P 2ac 2ab	Cell parameters from 4996 reflections
*a* = 14.2365 (5) Å	θ = 2.6–25.7º
*b* = 9.2098 (4) Å	µ = 0.21 mm^−^^1^
*c* = 26.3865 (10) Å	*T* = 295 (2) K
*V* = 3459.7 (2) Å^3^	Block, colourless
*Z* = 8	0.20 × 0.16 × 0.14 mm

### Data collection

**Table:**

Bruker Kappa APEXII diffractometer	4600 independent reflections
Radiation source: fine-focus sealed tube	2928 reflections with *I* > 2σ(*I*)
Monochromator: graphite	*R*_int_ = 0.034
*T* = 295(2) K	θ_max_ = 29.0º
ω and φ scans	θ_min_ = 1.5º
Absorption correction: multi-scan(SADABS; Sheldrick, 1996)	*h* = −19→19
*T*_min_ = 0.959, *T*_max_ = 0.971	*k* = −12→5
22287 measured reflections	*l* = −36→36

### Refinement

**Table:**

Refinement on *F*^2^	Secondary atom site location: difference Fourier map
Least-squares matrix: full	Hydrogen site location: inferred from neighbouring sites
*R*[*F*^2^ > 2σ(*F*^2^)] = 0.043	H-atom parameters constrained
*wR*(*F*^2^) = 0.144	*w* = 1/[σ^2^(*F*_o_^2^) + (0.0727*P*)^2^ + 0.3846*P*] where *P* = (*F*_o_^2^ + 2*F*_c_^2^)/3
*S* = 1.06	(Δ/σ)_max_ < 0.001
4600 reflections	Δρ_max_ = 0.33 e Å^−^^3^
244 parameters	Δρ_min_ = −0.31 e Å^−^^3^
Primary atom site location: structure-invariant direct methods	Extinction correction: none

### Fractional atomic coordinates and isotropic or equivalent isotropic displacement parameters (Å^2^)

**Table:**

	*x*	*y*	*z*	*U*_iso_*/*U*_eq_	
C1	0.97004 (12)	0.1799 (2)	0.69244 (7)	0.0401 (4)	
C2	1.04048 (14)	0.2223 (2)	0.72412 (9)	0.0533 (5)	
H2	1.0934	0.2681	0.7111	0.064*	
C3	1.03320 (16)	0.1971 (3)	0.77633 (9)	0.0604 (6)	
H3	1.0817	0.2255	0.7978	0.072*	
C4	0.95682 (15)	0.1322 (2)	0.79553 (8)	0.0534 (5)	
H4	0.9527	0.1177	0.8303	0.064*	
C5	0.88229 (13)	0.0854 (2)	0.76414 (7)	0.0422 (4)	
C6	0.80228 (14)	0.0161 (2)	0.78423 (8)	0.0515 (5)	
H6	0.7964	0.0055	0.8191	0.062*	
C7	0.73420 (15)	−0.0349 (3)	0.75369 (8)	0.0593 (6)	
H7	0.6819	−0.0804	0.7676	0.071*	
C8	0.74173 (14)	−0.0200 (3)	0.70142 (9)	0.0559 (5)	
H8	0.6950	−0.0579	0.6807	0.067*	
C9	0.81644 (13)	0.0493 (2)	0.68008 (7)	0.0463 (5)	
H9	0.8197	0.0595	0.6451	0.056*	
C10	0.88882 (11)	0.1055 (2)	0.71077 (6)	0.0380 (4)	
C11	1.06625 (13)	0.0248 (2)	0.58490 (7)	0.0427 (4)	
C12	1.10910 (12)	0.0642 (2)	0.53991 (7)	0.0399 (4)	
C13	1.08949 (12)	0.1663 (2)	0.49984 (6)	0.0410 (4)	
C14	1.01942 (14)	0.2671 (2)	0.49088 (7)	0.0484 (5)	
H14	0.9690	0.2756	0.5131	0.058*	
C15	1.02502 (17)	0.3552 (3)	0.44849 (8)	0.0625 (6)	
H15	0.9786	0.4240	0.4423	0.075*	
C16	1.0999 (2)	0.3407 (3)	0.41514 (8)	0.0716 (7)	
H16	1.1027	0.4011	0.3869	0.086*	
C17	1.16910 (19)	0.2414 (3)	0.42234 (8)	0.0677 (7)	
H17	1.2180	0.2317	0.3992	0.081*	
C18	1.16462 (14)	0.1546 (3)	0.46542 (7)	0.0520 (5)	
C19	1.19433 (14)	−0.0056 (3)	0.52798 (8)	0.0541 (5)	
C20	1.23384 (18)	−0.1090 (3)	0.55964 (10)	0.0730 (8)	
H20	1.2903	−0.1537	0.5513	0.088*	
C21	1.1879 (2)	−0.1435 (3)	0.60317 (11)	0.0749 (8)	
H21	1.2137	−0.2129	0.6247	0.090*	
C22	1.10310 (17)	−0.0774 (2)	0.61653 (8)	0.0588 (6)	
H22	1.0724	−0.1026	0.6464	0.071*	
N1	1.22599 (12)	0.0506 (2)	0.48255 (7)	0.0658 (6)	
H1	1.2766	0.0244	0.4673	0.079*	
O1	0.97919 (8)	0.08998 (15)	0.59681 (5)	0.0457 (3)	
O2	1.06505 (11)	0.30915 (17)	0.62191 (5)	0.0598 (4)	
O3	0.89333 (11)	0.30371 (18)	0.61460 (6)	0.0626 (4)	
S1	0.97877 (3)	0.23577 (6)	0.628849 (18)	0.04546 (16)	

### Atomic displacement parameters (Å^2^)

**Table:**

	*U*^11^	*U*^22^	*U*^33^	*U*^12^	*U*^13^	*U*^23^
C1	0.0447 (9)	0.0339 (10)	0.0415 (9)	−0.0010 (8)	0.0063 (7)	−0.0057 (7)
C2	0.0490 (11)	0.0476 (13)	0.0633 (12)	−0.0106 (9)	0.0029 (9)	−0.0084 (10)
C3	0.0636 (13)	0.0608 (16)	0.0568 (12)	−0.0069 (11)	−0.0150 (10)	−0.0117 (11)
C4	0.0691 (13)	0.0501 (13)	0.0410 (10)	0.0017 (11)	−0.0053 (9)	−0.0041 (9)
C5	0.0507 (10)	0.0343 (11)	0.0415 (9)	0.0051 (8)	0.0033 (8)	0.0004 (7)
C6	0.0607 (12)	0.0488 (13)	0.0452 (10)	0.0043 (10)	0.0121 (9)	0.0085 (9)
C7	0.0482 (12)	0.0624 (16)	0.0674 (14)	−0.0075 (10)	0.0138 (10)	0.0132 (11)
C8	0.0442 (11)	0.0612 (15)	0.0624 (12)	−0.0120 (10)	−0.0029 (9)	0.0051 (10)
C9	0.0447 (10)	0.0512 (13)	0.0432 (10)	−0.0032 (9)	0.0001 (8)	0.0022 (8)
C10	0.0389 (9)	0.0338 (10)	0.0414 (9)	0.0029 (8)	0.0046 (7)	−0.0014 (7)
C11	0.0463 (10)	0.0393 (11)	0.0426 (9)	−0.0011 (8)	−0.0025 (8)	−0.0088 (8)
C12	0.0386 (9)	0.0385 (11)	0.0425 (9)	−0.0004 (8)	−0.0005 (7)	−0.0118 (8)
C13	0.0425 (9)	0.0443 (12)	0.0362 (8)	−0.0069 (8)	0.0027 (7)	−0.0110 (8)
C14	0.0557 (11)	0.0483 (13)	0.0411 (10)	−0.0015 (10)	0.0005 (8)	−0.0048 (8)
C15	0.0870 (17)	0.0538 (15)	0.0466 (11)	−0.0035 (12)	−0.0115 (11)	−0.0014 (10)
C16	0.106 (2)	0.0670 (18)	0.0423 (11)	−0.0281 (16)	0.0004 (12)	−0.0027 (11)
C17	0.0813 (16)	0.0796 (19)	0.0423 (11)	−0.0303 (15)	0.0199 (11)	−0.0164 (11)
C18	0.0510 (11)	0.0589 (14)	0.0462 (10)	−0.0113 (10)	0.0086 (8)	−0.0190 (9)
C19	0.0454 (11)	0.0576 (14)	0.0594 (12)	0.0075 (10)	−0.0022 (9)	−0.0221 (10)
C20	0.0644 (15)	0.0709 (18)	0.0836 (17)	0.0301 (13)	−0.0190 (13)	−0.0302 (14)
C21	0.0970 (19)	0.0535 (16)	0.0741 (16)	0.0224 (14)	−0.0333 (15)	−0.0095 (12)
C22	0.0841 (16)	0.0438 (13)	0.0485 (11)	0.0015 (11)	−0.0115 (10)	−0.0025 (9)
N1	0.0471 (10)	0.0849 (16)	0.0656 (11)	0.0042 (10)	0.0176 (8)	−0.0262 (10)
O1	0.0445 (7)	0.0488 (9)	0.0439 (7)	−0.0075 (6)	0.0085 (5)	−0.0082 (6)
O2	0.0657 (10)	0.0492 (10)	0.0646 (9)	−0.0207 (8)	0.0269 (7)	−0.0099 (7)
O3	0.0674 (10)	0.0622 (11)	0.0582 (9)	0.0180 (8)	0.0182 (7)	0.0154 (7)
S1	0.0501 (3)	0.0403 (3)	0.0459 (3)	−0.0033 (2)	0.0158 (2)	−0.0008 (2)

### Geometric parameters (Å, °)

**Table:**

C1—C2	1.363 (3)	C12—C13	1.442 (3)
C1—C10	1.429 (2)	C13—C14	1.383 (3)
C1—S1	1.7594 (19)	C13—C18	1.407 (2)
C2—C3	1.401 (3)	C14—C15	1.384 (3)
C2—H2	0.9300	C14—H14	0.9300
C3—C4	1.340 (3)	C15—C16	1.388 (3)
C3—H3	0.9300	C15—H15	0.9300
C4—C5	1.413 (3)	C16—C17	1.358 (4)
C4—H4	0.9300	C16—H16	0.9300
C5—C6	1.409 (3)	C17—C18	1.391 (3)
C5—C10	1.424 (2)	C17—H17	0.9300
C6—C7	1.345 (3)	C18—N1	1.373 (3)
C6—H6	0.9300	C19—N1	1.381 (3)
C7—C8	1.390 (3)	C19—C20	1.386 (3)
C7—H7	0.9300	C20—C21	1.359 (4)
C8—C9	1.362 (3)	C20—H20	0.9300
C8—H8	0.9300	C21—C22	1.398 (3)
C9—C10	1.409 (2)	C21—H21	0.9300
C9—H9	0.9300	C22—H22	0.9300
C11—C22	1.363 (3)	N1—H1	0.8600
C11—C12	1.383 (3)	O1—S1	1.5867 (14)
C11—O1	1.413 (2)	O2—S1	1.4139 (15)
C12—C19	1.409 (3)	O3—S1	1.4186 (15)
			
C2—C1—C10	121.70 (18)	C18—C13—C12	106.01 (17)
C2—C1—S1	116.71 (15)	C13—C14—C15	119.3 (2)
C10—C1—S1	121.35 (13)	C13—C14—H14	120.3
C1—C2—C3	120.09 (19)	C15—C14—H14	120.3
C1—C2—H2	120.0	C14—C15—C16	120.0 (2)
C3—C2—H2	120.0	C14—C15—H15	120.0
C4—C3—C2	120.4 (2)	C16—C15—H15	120.0
C4—C3—H3	119.8	C17—C16—C15	122.2 (2)
C2—C3—H3	119.8	C17—C16—H16	118.9
C3—C4—C5	121.59 (19)	C15—C16—H16	118.9
C3—C4—H4	119.2	C16—C17—C18	117.9 (2)
C5—C4—H4	119.2	C16—C17—H17	121.0
C6—C5—C4	121.64 (18)	C18—C17—H17	121.0
C6—C5—C10	118.94 (17)	N1—C18—C17	129.9 (2)
C4—C5—C10	119.40 (17)	N1—C18—C13	108.96 (18)
C7—C6—C5	121.01 (19)	C17—C18—C13	121.2 (2)
C7—C6—H6	119.5	N1—C19—C20	130.4 (2)
C5—C6—H6	119.5	N1—C19—C12	107.7 (2)
C6—C7—C8	120.28 (19)	C20—C19—C12	121.9 (2)
C6—C7—H7	119.9	C21—C20—C19	118.3 (2)
C8—C7—H7	119.9	C21—C20—H20	120.8
C9—C8—C7	121.1 (2)	C19—C20—H20	120.8
C9—C8—H8	119.4	C20—C21—C22	121.8 (2)
C7—C8—H8	119.4	C20—C21—H21	119.1
C8—C9—C10	120.37 (18)	C22—C21—H21	119.1
C8—C9—H9	119.8	C11—C22—C21	118.6 (2)
C10—C9—H9	119.8	C11—C22—H22	120.7
C9—C10—C5	118.22 (16)	C21—C22—H22	120.7
C9—C10—C1	125.02 (16)	C18—N1—C19	109.83 (16)
C5—C10—C1	116.75 (16)	C18—N1—H1	125.1
C22—C11—C12	122.49 (19)	C19—N1—H1	125.1
C22—C11—O1	119.68 (18)	C11—O1—S1	118.79 (11)
C12—C11—O1	117.78 (17)	O2—S1—O3	119.98 (11)
C11—C12—C19	116.86 (19)	O2—S1—O1	109.40 (8)
C11—C12—C13	135.65 (17)	O3—S1—O1	103.61 (9)
C19—C12—C13	107.48 (17)	O2—S1—C1	108.93 (9)
C14—C13—C18	119.31 (19)	O3—S1—C1	108.73 (9)
C14—C13—C12	134.63 (16)	O1—S1—C1	105.14 (8)
			
C10—C1—C2—C3	−1.9 (3)	C15—C16—C17—C18	−1.5 (3)
S1—C1—C2—C3	172.46 (18)	C16—C17—C18—N1	−177.6 (2)
C1—C2—C3—C4	−0.5 (4)	C16—C17—C18—C13	1.6 (3)
C2—C3—C4—C5	1.0 (4)	C14—C13—C18—N1	178.73 (17)
C3—C4—C5—C6	179.5 (2)	C12—C13—C18—N1	0.9 (2)
C3—C4—C5—C10	0.9 (3)	C14—C13—C18—C17	−0.6 (3)
C4—C5—C6—C7	−176.4 (2)	C12—C13—C18—C17	−178.51 (18)
C10—C5—C6—C7	2.2 (3)	C11—C12—C19—N1	−178.93 (17)
C5—C6—C7—C8	0.0 (4)	C13—C12—C19—N1	−0.1 (2)
C6—C7—C8—C9	−1.6 (4)	C11—C12—C19—C20	0.0 (3)
C7—C8—C9—C10	1.0 (3)	C13—C12—C19—C20	178.8 (2)
C8—C9—C10—C5	1.2 (3)	N1—C19—C20—C21	179.0 (2)
C8—C9—C10—C1	−179.92 (19)	C12—C19—C20—C21	0.3 (4)
C6—C5—C10—C9	−2.7 (3)	C19—C20—C21—C22	−0.2 (4)
C4—C5—C10—C9	175.89 (18)	C12—C11—C22—C21	0.7 (3)
C6—C5—C10—C1	178.26 (17)	O1—C11—C22—C21	178.11 (18)
C4—C5—C10—C1	−3.1 (3)	C20—C21—C22—C11	−0.3 (4)
C2—C1—C10—C9	−175.26 (19)	C17—C18—N1—C19	178.3 (2)
S1—C1—C10—C9	10.6 (3)	C13—C18—N1—C19	−1.0 (2)
C2—C1—C10—C5	3.7 (3)	C20—C19—N1—C18	−178.2 (2)
S1—C1—C10—C5	−170.45 (14)	C12—C19—N1—C18	0.6 (2)
C22—C11—C12—C19	−0.6 (3)	C22—C11—O1—S1	91.6 (2)
O1—C11—C12—C19	−177.99 (16)	C12—C11—O1—S1	−90.91 (17)
C22—C11—C12—C13	−179.0 (2)	C11—O1—S1—O2	25.23 (16)
O1—C11—C12—C13	3.6 (3)	C11—O1—S1—O3	154.30 (13)
C11—C12—C13—C14	0.7 (4)	C11—O1—S1—C1	−91.63 (14)
C19—C12—C13—C14	−177.9 (2)	C2—C1—S1—O2	4.21 (19)
C11—C12—C13—C18	178.1 (2)	C10—C1—S1—O2	178.61 (15)
C19—C12—C13—C18	−0.5 (2)	C2—C1—S1—O3	−128.18 (17)
C18—C13—C14—C15	−0.6 (3)	C10—C1—S1—O3	46.22 (18)
C12—C13—C14—C15	176.6 (2)	C2—C1—S1—O1	121.38 (16)
C13—C14—C15—C16	0.8 (3)	C10—C1—S1—O1	−64.22 (16)
C14—C15—C16—C17	0.3 (4)		

### Hydrogen-bond geometry (Å, °)

**Table:**

*D*—H···*A*	*D*—H	H···*A*	*D*···*A*	*D*—H···*A*
C2—H2···O2	0.93	2.42	2.835 (3)	107
C8—H8···O3^i^	0.93	2.50	3.403 (3)	164
C17—H17···O3^ii^	0.93	2.54	3.364 (3)	147

Symmetry codes: (i) −*x*+3/2, *y*−1/2, *z*; (ii) *x*+1/2, −*y*+1/2, −*z*+1.
